# Defect-free high Sn-content GeSn on insulator grown by rapid melting growth

**DOI:** 10.1038/srep38386

**Published:** 2016-12-12

**Authors:** Zhi Liu, Hui Cong, Fan Yang, Chuanbo Li, Jun Zheng, Chunlai Xue, Yuhua Zuo, Buwen Cheng, Qiming Wang

**Affiliations:** 1 State Key Laboratory on Integrated Optoelectronics, Institute of Semiconductors, Chinese Academy of Sciences, Beijing 100083, People’s Republic of China

## Abstract

GeSn is an attractive semiconductor material for Si-based photonics. However, large lattice mismatch between GeSn and Si and the low solubility of Sn in Ge limit its development. In order to obtain high Sn-content GeSn on Si, it is normally grown at low temperature, which would lead to inevitable dislocations. Here, we reported a single-crystal defect-free graded GeSn on insulator (GSOI) stripes laterally grown by rapid melting growth (RMG). The Sn-content reaches to 14.2% at the end of the GSOI stripe. Transmission electron microscopy observation shows the GSOI stripe without stacking fault and dislocations. P-channel pseudo metal-oxide-semiconductor field effect transistors (MOSFETs) and metal-semiconductor-metal (MSM) Schottky junction photodetectors were fabricated on these GSOIs. Good transistor performance with a low field peak hole mobility of 402 cm^2^/Vs is obtained, which indicates a high-quality of this GSOI structure. Strong near-infrared and short-wave infrared optical absorption of the MSM photodetectors at 1550 nm and 2000 nm were observed. Owing to high Sn-content and defect-free, responsivity of 236 mA/W@-1.5 V is achieved at 1550 nm wavelength. In addition, responsivity reaches 154 mA/W@-1.5 V at 2000 nm with the optical absorption layer only 200 nm-thick, which is the highest value reported for GeSn junction photodetectors until now.

GeSn is an attractive semiconductor for Si-based photonics due to its special energy band structure and the compatibility with complementary metal oxide semiconductor (CMOS) processes^1^,^2^,^3^,^4^,^5^,^6^,^7^,^8^. GeSn with tunable direct bandgap from 0.8 eV to 0 eV has great potential in developing Si-based infrared photodetectors. Si-based GeSn photodetectors have been achieved working at near-infrared^5^,^6^ and short-wave infrared wavelength range^7^,^8^, which are mostly dominated by direct-bandgap group III-V materials. Meanwhile, GeSn also is the most promising material for achieving efficient Si-based group IV light source. Several experimental results have proven that GeSn alloy would transform from an indirect bandgap semiconductor to a direct one when the Sn-content increases to approximately 7–8%^1^,^2^. Si-based light emitting diodes^3^ and an optical pumping laser from direct bandgap GeSn^4^ have been reported. However, due to the large lattice constant, high Sn-content GeSn layer grown on Si substrate usually has the poor material quality and under unexpected compressive strain. Compressive strain increases the energy difference between *Γ* valley and *L* valley in conduction band of GeSn, and offsets the bandgap shrink induced by Sn incorporation, which prevents direct bandgap transformation and high absorption coefficient^4^,^5^,^7^,^8^,^9^. For these reasons, the key to enhance the optical absorption and light emission performance is not only promoting the Sn-content and crystal quality, but also relaxing the compressive strain (even obtains tensile strain) of GeSn layer.

However, the epitaxial growth of high Sn-content GeSn has several difficulties: (1) Sn tends to segregate to the surface of GeSn; (2) the equilibrium solid solubility of Sn in Ge is rather low (<1%); (3) there is a large lattice mismatch of 14.7% (19.5%) between α-Sn and Ge (Si). Despite these difficulties, significant efforts have been made in growing GeSn by chemical vapor deposition (CVD)^4^,^7^,^10^ and molecular beam epitaxy (MBE)^1^,^11^,^12^,^13^ under non-equilibrium condition at low temperature, which is used to suppress segregation of Sn. In order to increase Sn-content in GeSn layer, grown temperature has to reduce further, which could be achieved using MBE. The grown temperature even was lower than 160 °C using MBE to obtain high Sn-content GeSn layer^11^,^12^. However, due to the combination effects of low adatom mobility and the presence of Ehrlich barriers at descending step edges^14^, low temperature grown technology also brings some problems, such as Kinetic surface roughening^15^, poor quality, and even epitaxial breakdown (amorphization)^11^,^16^. Meanwhile, large lattice mismatch induced by Sn incorporation also is an inevitable problem at low grown temperature. Fully strained GeSn layer without dislocation can be grown within a restricted critical thickness. When the thickness of GeSn layer is larger than critical thickness, misfit dislocations and threading dislocations formed in the film, which may restrict the application of relaxed GeSn layers in microelectronics and optoelectronics^17^,^18^. High crystal-quality, high Sn-content, and strain relaxation are contradictory to each other in GeSn layer grown on Si substrate. Therefore, it is a great challenge to grow strain relaxed, high-quality, and high Sn-content GeSn.

Recently, a technique (RMG), similar to Czochralski crystal growth process, about fabricating defect-free Ge on insulator (GOI) has been described^19^,^20^, in which single-crystal Si was used as a crystal seed for lateral liquid phase epitaxial growth. Like a ‘necking effect’ in nanoscale selective epitaxial growth process^21^, this technique enables to terminate the lattice mismatch and dislocation in sidewalls, and obtains defect-free single-crystal GOI stripes. Ge avalanche photodetectors fabricated by this method were integrated on silicon on insulator wafers operated at 30 Gbps by standard CMOS processing^22^. Moreover, without constraint from substrate lattice, those free GOI stripes are almost strain relaxed except a small thermal tensile strain (<0.3%) induced by expansion difference between Ge and Si substrate^23^,^24^. Therefore, this technique has potential in grown defect-free and tensile strained GSOI stripes. Tensile strained single-crystal GSOI stripes grown by this method with graded Sn-content have been demonstrated^25^,^26^. However, Sn-content in these GSOI is lower than 6%.

In this work, defect-free GSOI with high Sn-content was laterally grown on an insulator by RMG. High initial Sn-content in amorphous GeSn film and high cooling rate in cooling process were used to increase the Sn-content of the GSOI. The gradient of Sn-content with the highest Sn-content of 14.2% was created along the GeSn strips by growing. No stacking fault and no dislocation were observed in the GSOI stripe by TEM. Electrical and optical characteristics of the GSOIs were studied by pseudo-MOSFETs and MSM Schottky junction photodetectors. High hole mobility of 402 cm^2^/Vs is obtained. Responsivity reaches 154 mA/W@-1.5 V at 2000 nm with the optical absorption layer only 200 nm, which is the highest result reported for GeSn junction photodetectors until now.

## Results

### Crystal orientation, Sn-content, and Sn distribution of GSOI stripe

**Figure 1:**
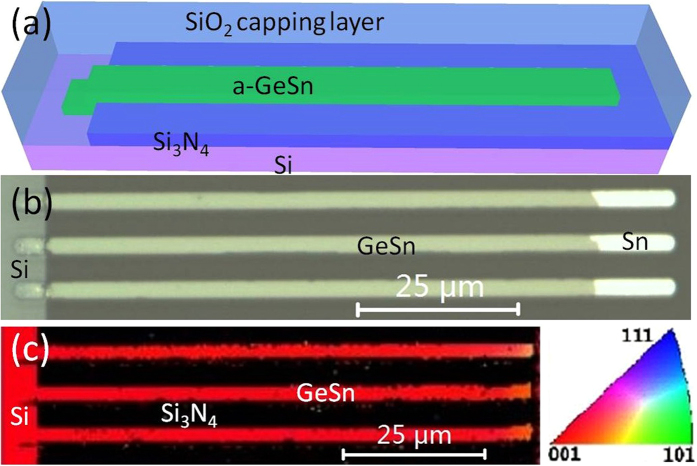
(**a**) Schematic of sample structure before RMG. The amorphous GeSn stripe has a head contact with the Si substrate though seed window. The width, length, and thickness of the GeSn stripe are 2.2–2.7 μm, 10–200 μm, and 160–200 nm, respectively. (**b**) Top-view optical micrograph of GSOIs after RMG with 89 μm in length. The initial length of the stripes in grown region is 100 μm. Because of the Sn segregation, actual length of GSOI stripe reduced to 89 μm after RMG. (**c**) EBSD image of 89 μm-length GSOI stripes. (001) orientation and Si3N4 area exhibit red and dark color, respectively.

Figure 1(a) is a schematic of sample structure before RMG. Figure 1(b) shows a top-view optical micrograph of the 89 μm-length GSOIs after RMG. At the end of the GSOI stripes, white bright metal Sn stripes were observed. It indicated that the segregation of Sn atoms occurred during the RMG due to the low equilibrium solid solubility of Sn in Ge. The Sn atoms were segregated, collected, and pushed to the end of the GSOI stripe by the lateral growth process of the GeSn. This Sn precipitation at the end of the stripe confirms the accomplishment of lateral growth. After removed the SiO_2_ capping layer, crystal orientation was measured by electron backscattering diffraction (EBSD). In EBSD image (Fig. 1(c)), except a lattice rotation at the end of the GSOI stripes, most part of the GSOI stripes exhibits (001) single-crystal orientation (red) as same as the Si (001) substrate. This typical RMG orientation indicates that the GSOI stripe has undergone a lateral growth process from Si seed.

**Figure 2:**
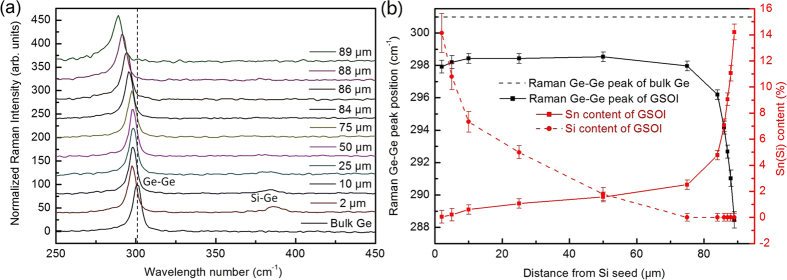
(**a**) Typical micro-Raman spectra of a GSOI stripe with 89 μm in length. Bulk Ge with the Ge-Ge mode peak at 301 cm^−1^ is also shown for comparison. (**b**) The peak positions of Ge-Ge Raman mode (dark curve), Si-content (red circle), and Sn-content (red square) of the GSOI stripe in different positions. Bulk Ge with the Ge-Ge mode peak at 301 cm^−1^ (dark dash line) is also shown for comparison.

Micro-Raman measurements were used to evaluate the lateral profiles of Si, Ge, and Sn concentrations in the GSOI stripe. Figure. 2(a) shows typical micro-Raman spectra obtained from different positions of a GSOI stripe. The main-peaks (288–300 cm^−1^) attributed to the Ge-Ge vibration mode, are observed in all spectra. The Si-Ge sub-peaks (~380 cm^−1^) induced by Si-Ge mixing and diffusion are observed in the region near the seeding region (0–50 μm). This intense lateral Si diffusion is the typical feature of completely melting of the GeSn stripe. A remarkable Ge-Ge mode shift can be observed along the GSOI stripe grown direction. Firstly, Ge-Ge mode shifts to high-frequency with the position near the Si seed (0–50 μm). This shift is mainly induced by the Si-content decreasing along the GSOI stripe, which is in tallies with appearance of the Si-Ge peak. Secondly, Ge-Ge mode peak shifts to low-frequency at the position far from the seeding region (50–89 μm) monotonously. It is mainly attributed to the GeSn alloys by incorporation of Sn into Ge. At the end of the GSOI stripe (89 μm), an outstanding low-frequency shift extend to 12.5 cm^−1^ compared with bulk Ge, which indicates the high Sn-content of the GSOI. The peak positions of Ge-Ge mode in Raman spectra of the GSOI stripe are summarized in Fig. 2(b), as a function of distance from the Si seed.

The Sn-content (*x*) of Si_y_Ge_1-x-y_Sn_x_ stripe with a low Si content could be calculated from the following equation^27^,^28^:


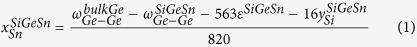


where 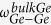 and 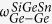 are the wave number of the Ge-Ge Raman peak of bulk Ge and SiGeSn film, respectively. 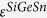 is the strain of SiGeSn. To determine the Sn-content of the GSOI accurately, low-frequency shift induced by thermal tensile and Si-content strain should be taken into account. Usually, thermal tensile strain induced by expansion difference between GeSn (Ge) and Si substrate exists in whole stripe^23^,^24^,^25^,^26^. Thus, in the region with Si-content (0–50 μm), the low-frequency shift is induced by tensile strain, Si-content, and Sn-content. With the Si-content disappearance at back region (50–89 μm), the low-frequency shift is attributed to tensile strain and Sn-content. The Si-content (*y*) of GOI stripes can be calculated from the ratio between the integrated intensities of the Raman peaks corresponding to the Ge-Ge bonds and Ge-Si bonds^29^. The calculational Si-content distribution along the GSOI stripe at different positions also shows in Fig. 2(b). The Si-content and related calculational details of GSOI stripes with various lengths can be found in supplementary material. After excluding the low-frequency shift induced by Si-content, a low-frequency shift of 1.1 ± 0.2 cm^−1^ induced by tensile strain and Sn-content is obtained at the GSOI stripe near the Si seed (<25 μm), where has the lowest Sn-content in whole GSOI stripe. Based on our previous GSOI work, Sn-content is about 0.3% (equilibrium solid solubility of Sn in Ge) near initial nucleic position^30^, which also coincides with the GSOI RMG report^25^. Thus Sn-content of 0.3% is used for the GSOI stripe near the Si seed. After remove the low-frequency shift of 0.25 cm^−1^ induced by Sn-content of 0.3%, tensile strain of the GSOI can be calculated to be about 0.15% at the stripe near the Si seed. We suppose the tensile strain is uniform at whole GSOI stripe. Due to the small tensile strain (<0.3%), Sn-content error of the GOIs induced by this supposition would lower than 1%. After getting the Si-content and tensile strain of the GSOI, Sn-content distribution along the GSOI stripe is calculated and shown in Fig. 2(b). The Sn-content of the GSOI increases monotonously as the distance from the Si seed. Especially, a dramatic Sn-content increase occurred at the end of the GSOI, with the highest Sn-content about 14.2%. Sn-content distribution in GSOI can be simulated by Scheil equation^31^. Sn-content simulation curves and Sn content distribution of the GSOI stripe with various lengths are shown in supplementary material. When the length of the stripe is shorter than 89 μm, the highest Sn-content of the GSOI strongly depends on the length of the stripe, and raises as the length of GSOI increasing.

**Figure 3:**
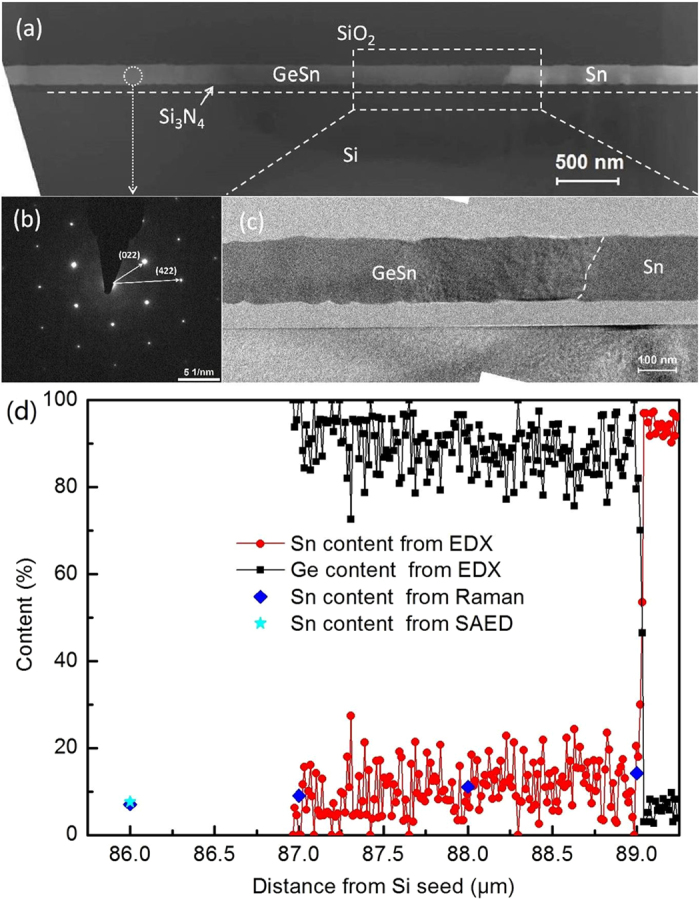
(**a**) STEM image of the GSOI stripe near the GeSn/Sn interface (85 μm–90.5 μm away from Si seed). The thickness and length of the GSOI stripe are 160 nm and 89 μm, respectively. Bright metal Sn and GeSn/Sn interface is clearly observed at the end of the GSOI stripe. (**b**) SAED pattern of the GSOI stripe at 3 μm away from the GeSn/Sn interface (86 μm away from Si seed). (**c**) TEM image of the GSOI near the GeSn/Sn interface. A clear Si_3_N_4_/Si interface was observed. The thickness of the Si_3_N_4_ layer is 63 nm. (**d**) The Sn-content and Ge-content distributions of the GSOI stripe within 2 μm length near the GeSn/Sn interface by EDX under the TEM model. Sn-content by Raman results (blue diamond), and Sn-content by SAED (cyan star) are shown for comparison.

To clarify the crystal quality and structure of the GSOI stripe, cross-sectional transmission electron microscopy (TEM) was carried out near the GeSn/Sn interface for a GSOI with 89 μm in length. Figure 3(a) and Fig. 3(c) show a scanning transmission electron microscopy (STEM) and TEM micrographs of the GSOI, respectively. Bright metal Sn is clearly observed at the end of the GSOI. Due to the “neck bottle effecting” and thin thickness of the GSOI structure, no stacking fault and no dislocation defect is observed. A selected area electron diffraction (SAED) pattern of the GSOI stripe is shown in Fig. 3(b) at 3 μm away from the GeSn/Sn interface. According to the SAED pattern, lattice constant of the GSOI at the diffraction position is 5.732 ± 0.003 Å, which corresponds to the Sn-content of 7.6 ± 0.3%^10^. This Sn-content coincides with that obtained from the micro-Raman spectra (7.1%). Sn and Ge content profiles also were estimated from the energy dispersive X-ray spectroscopy (EDX) under the TEM model for this GSOI within 2 μm length near the GeSn/Sn interface (Fig. 3(d)). Like the micro-Raman results, Sn-content of the GSOI stripe reduces as the distance far from the GeSn/Sn interface. Sn-content from micro-Raman and SAED are also shown in Fig. 3(d). All Sn-content results are in good agreement with each other, which indicates that high Sn-content GSOI with the Sn-content larger than 14% is achieved successfully.

### Electrical measurements

**Figure 4:**
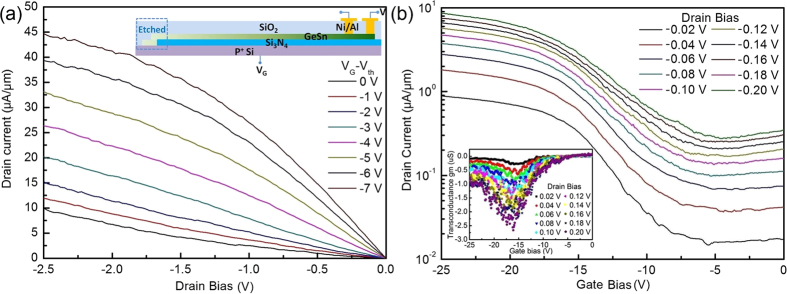
(**a**) I_D_-V_D_ characteristics of the device at gate bias higher than V_th_ ranging from 0 V to −7 V in −1 V step. The inset is the schematic of the back-gate transistor. (**b**) I_D_-V_G_ characteristics of the device with drain bias ranging from −0.02 V to −0.2 V in −0.02 V step. The inset is transconductance (g_m_) curves of the GSOI pseudo-MOSFET of the device with drain bias ranging from −0.02 V to −0.2 V. The average Sn concentration, thickness, width, and length of the GeSn channel are 8.5%, 160 nm, 2.7 μm, and 3.8 μm, respectively.

After etching heads of the stripes to disconnect contact to Si substrate, back-gate pseudo-MOSFETs were fabricated to study the electrical characteristics of the GSOI stripe. Schematic structure of the GSOI pseudo-MOSFET is shown in the inset of Fig. 4(a). A 63 nm-thick Si_3_N_4_ layer between the GeSn stripe and the p^+^ Si substrate was used as a gate dielectric. The width (length) of the GeSn channel is 2.7 μm (3.8 μm), which is obtained by scanning electron microscopy (SEM). The average Sn-content of the channel is about 8.5%. The *I*_D_-*V*_D_ characteristics of the device with gate bias ranging from 0 V to −7 V are shown in Fig. 4(a). The threshold voltage (*V*_th_) of the pseudo-MOSFET is about −12 V. The device exhibits a good p-type FET operation with a hole inversion mode. The linear, nonlinear, and saturation regions can be observed clearly. The drain current increases fast at high drain bias, which could be attributed to the short channel distance (3.8 μm) and soft breakdown leakage current from Schottky junction under high electric field.

The *I*_D_-*V*_G_ characteristics of the device are shown in Fig. 4(b) with drain bias ranging from −0.02 V to −0.2 V. A good switching performance is obtained with on/off current ratio about 10^2^. The off-leakage current is about 10^−2^ μA/μm. According to the *I*_D_-*V*_G_ characteristics, the initial conductivity type of the GSOI is n-type obviously. Therefore, the off-leakage current is dominated by the body leakage current of the thick GeSn (160 nm). The low-field hole mobility *μ*_0_ can be extracted from the I_D_-V_G_ characteristics by the following equation^32^:


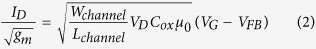


where *g*_m_ is the transconductance, *C*_ox_ is the capacitance of the gate dielectric, and *V*_FB_ is the flatband voltage estimated from the I_D_-V_G_ curve. *C*_ox_ = 1.06 × 10^−7^ F/cm^2^ was obtained by the capacitance-voltage (C-V) measurement. Transconductance curves of the GSOI pseudo-MOSFET are shown in the inset of Fig. 4(b) with drain bias ranging from −0.02 V to −0.2 V. The peak hole mobility of the GSOI pseudo-MOSFET is about 402 cm^2^/Vs under drain bias of −0.1 V with the gate bias around −18 V, much higher than that of common Si p-channel MOSFETs (~180 cm^2^/Vs)^33^ and Ge p-channel MOSFETs (~270 cm^2^/Vs)^34^. The 0.15% tensile strain is too small to enhance the mobility. Thus, the hole mobility enhancement mainly induced by defect-free high Sn-content of GeSn and good quality of GeSn/Si_3_N_4_ interface.

### Optical responsivity measurements

**Figure 5:**
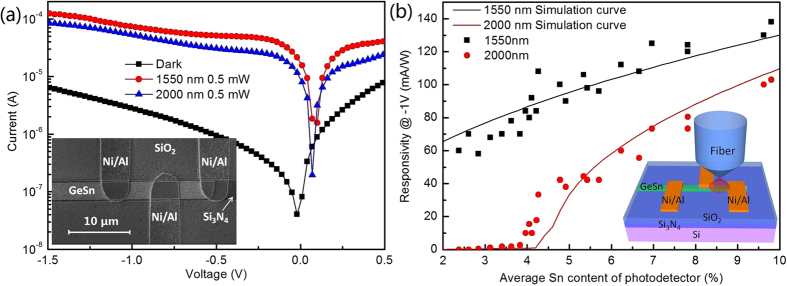
(**a**) I-V characteristics of a MSM Schottky junction photodetector with an average Sn-content of 9.8% at the light-receiving area. The thickness of the GeSn layer is 200 nm. The light-receiving area of the GSOI is only 2.7 × 3.8 μm^2^. A 1550 nm and 2000 nm incident light were introduced by a tapered lensed fiber with a light power of 0.5 mW. The diameter of the incident light beam is about 4–7 μm, which is much larger than the light-receiving area of the GSOI. The inset is SEM image of the device. At the end of GeSn stripe, Ni/Al electrodes and Si_3_N_4_ layer are observed. Si_3_N_4_ layer under the metal Sn was exposed by HF wet etching, which was used to open the electrode’s contact holes. (**b**) The 1550 nm and 2000 nm optical responsivity of devices with various Sn-content at −1 V. The average Sn-content range of different devices is 2.4–9.8%. Optical responsivity simulation curves calculated based on bandgap of the GeSn alloy with various Sn-content are also shown for comparison. The inset is a schematic of the optical responsivity measurement.

The band structure of GeSn alloy is very sensitive to the Sn-content. The pseudo-MOSFET structure also can work as a MSM photo-detector to study the optical characteristics of the high Sn-content GSOI. The current-voltage (I-V) characteristics and SEM image of a device are shown in Fig. 5(a). The device exhibits an obvious rectifying behavior with a low dark current of 2.8 μA (6.4 μA) at −1 V (−1.5 V). This rectifying behavior indicates that Schottky junctions have different barrier height at the GeSn/Ni contacts. The higher barrier is at the contact between low Sn-content GeSn and Ni, which is confirmed by I-V measurements. The dark current depends on the Sn-content of the GSOI and increase as the Sn-content increasing, which is attributed to bandgap shrink in the high Sn-content GSOI. The I-V characteristic of the devices with different average Sn-content can be found in supplementary material. The inset of Fig. 5(b) shows the schematic of the optical responsivity measurement. The device has optical responsivity at 0 V, which indicates the device operates at Schottky junction mode. Incident light with wavelength of 1550 nm or 2000 nm was introduced by a tapered lensed fiber with a light power of 0.5 mW. The diameter of the incident light beam is about 4–7 μm, which is larger than the light-receiving area of the device. However, a strong optical absorption at 1550 nm and 2000 nm is observed. Large photocurrents of 69 μA (51 μA) and 118 μA (77 μA) are obtained at −1 V and −1.5 V at 1550 nm (2000 nm), respectively. The responsivity of the device at 1550 nm (2000 nm) is 138 mA/W@-1V (102 mA/W@-1V) and 236 mA/W@-1.5 V (154 mA/W@-1.5 V). Although the optical responsivity of the device is underestimated due to the small light-receiving area, the responsivity at 2000 nm is the highest for Si-based GeSn photodetector so far. If compared with the GeSn photodetector with same absorption layer, the responsivity at 1550 nm also is the highest result for GeSn photodetector. Comparison of responsivity of GeSn top-illuminated photodetectors can be found in supplementary material. This high optical responsivity benefited from the defect-free high Sn-content GSOI with small tensile strain. GeSn PIN photodetector with high Sn-content up to 10% has been reported^7^. However, due to the inevitable dislocations of the high Sn-content GeSn layers grown on Si substrate, the performance of the photodetector is not satisfactory even lower than that of device with low Sn-content^7^. Meanwhile, our GSOI is under 0.15% tensile strain, but all of the Si-based GeSn photodetectors are under large unexpected compressive strain, which increase the bandgap and reduces the absorption coefficient^5^,^7^,^8^.

To study the band edge absorption effect of the GSOIs, the optical responsivity of devices with various average Sn-content were studied. The 1550 nm and 2000 nm optical responsivity of devices at −1 V are shown in Fig. 5(b) with various Sn-content. Because of GSOI/electrode contact, the high Sn-content region (~14.2%) was covered by metal electrode at the end of GeSn stripe. Therefore, the highest average Sn concentration of devices is decreased. Here, the average Sn-content range of different devices is 2.4–9.8%. All devices exhibit a strong optical responsivity at 1550 nm. The optical responsivity raises as the average Sn-content increasing, which can be explained by the absorption coefficient increase. When the incident light wavelength is 2000 nm, the optical responsivity of devices is totally different with that at 1550 nm. When the average Sn-content lower than 3%, no obvious optical responsivity is observed, which indicates neither indirect bandgap energy nor direct bandgap energy of GeSn are smaller than 0.62 eV (2000 nm). As the average Sn-content is between 3–4%, a weak optical absorption is observed, which is most likely induced by an indirect band absorption. Strong optical responsivity is obtained as Sn-content is larger than 4%, and rises quickly as the Sn-content increasing. This optical responsivity is attributed to the direct band optical absorption of the GSOI.

To describe the optical responsivity behaviors of the devices accurately, the optical responsivity can be expressed as^7^:


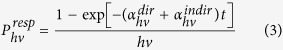


where *hν* is the incident photon energy, 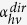 and 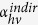 are the absorption coefficients which attribute to the light absorption from direct band and indirect band, respectively. Near the bandgap, the absorption coefficient can be expressed as following equations with the photon energy comparable with the bandgap energy^7^,^35^:


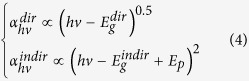



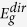
 is the direct bandgap energy, 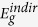 is the indirect bandgap energy of the material, *t* is the thickness of the material, *E*_p_ is the energy of the phonon that allows indirect bandgap transition. The equation of 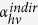 is a empirical formula. Usually, 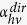 is about two or three orders of magnitude larger than 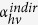. Most of light absorption is induced by direct band absorption. Although, 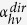 and 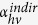 are not in the same unit in the formulas, the ratio of 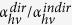 is reasonable. The band structure of the GeSn alloy was calculated using the deformation potentials theory^36^. Bandgap schematic diagrams and related calculational details of GeSn with various Sn-content can be found in supplementary material. Because the valence band is split by strain, bandgap between light hole valence band (LHVB) edge and conduction band (CB) is smaller than that of heavy hole valence band (HHVB). The calculated optical responsivity based on those bandgap is shown in Fig. 5(b) for comparison. The simulation curve (1550 nm) raises as the Sn-content increasing, which is in good agreement with the experiment results. For the simulation curve with wavelength of 2000 nm, an obvious optical absorption start at the Sn-content of 3%, which is as same as the experimental results. The optical absorption is dominated by indirect band transition (3–4.2% Sn-content), LHVB edge to CB transition (4.2–4.6% Sn-content), and both LHVB/HHVB edges to CB transition (>4.6% Sn-content) are exhibited clearly. This simulation curve fitted the experiment results good at the average Sn-content larger than 5%. However, a difference between the simulation curve and experimental results is found at the average Sn-content among 4–5%. GeSn alloy with Sn-content of 4–5% has a direct bandgap energy of 0.62 ± 0.02 eV, and very closed to the photon energy (0.62 eV). Due to the direct bandgap transition it has a much larger absorption coefficient than that of indirect bandgap transition. When the direct bandgap is close to the incident photon energy, light absorption is very sensitive to the small change in Sn-content. Thus, this difference could be explained by the gradient of Sn-content in the GSOI. According to the Sn distribution in the GSOI, the highest Sn-content of the light-receiving area is about 10–15% larger than the average Sn concentration. Most of the optical absorption should origin from highest Sn-content region of the light-receiving area.

## Discussion

In summary, RMG is a potential technique in growing defect-free and tensile strained GSOI stripes with high Sn-content. A defect-free high Sn-content GSOI was laterally grown by RMG. EBSD results confirmed a single-crystal orientation of the GSOIs. The gradient of Sn-content was created along the GSOI by growing process, which was in agreement with calculated results based on Scheil equation. The highest Sn-content at the end of GSOI strongly depends on the length of the GSOI stripe, and it achieves 14.2%, which is confirmed by micro-Raman, EDX, and TEM. P-channel pseudo-MOSFETs and MSM photodetectors were fabricated on the GSOIs to study the optical and electrical characteristics of the GSOIs. Good transistor performance with the low field peak hole mobility of 402 cm^2^/Vs is obtained, which indicates a high-quality of the GSOI structure. This high hole mobility also indicates that the defect-free high Sn-content GSOI is a good potential structure to fabricate high-speed MOSFET, which enabled monolithic integration of GeSn transistors with Si transistors on Si substrate for three-dimensional Si large-scale integrated circuits. Strong optical absorption of the MSM photodetectors at 1550 nm and 2000 nm was observed. Optical absorptions origin from indirect band absorption and direct band absorption of the MSM photodetectors by Sn incorporation were clearly observed. The highest optical responsivity of 236 mA/W and 154 mA/W for the device (average Sn-content of 9.8%) were achieved at −1.5 V with the wavelength of 1550 nm and 2000 nm, respectively. This optical responsivity at 2000 nm is the highest value for GeSn junction photodetectors, with the optical absorption layer only 200 nm. These results indicate that this GSOI is also a good potential structure to fabricate high optical responsivity photodetectors cover near-infrared, short-wave infrared, and mid-infrared. The highest Sn-content of the GSOI with 0.15% tensile strain is 14.2%, which is high enough to transform the GeSn to a direct bandgap semiconductor, which also is a good potential semiconductor for high efficiency light resources and even GeSn laser.

## Methods

### Fabrication of the GSOI structures

A 63 nm Si_3_N_4_ layer was grown on p^+^ Si (001) substrates with a resistivity of 0.001 Ω-cm using low pressure chemical vapor deposition (LPCVD) followed by photolithography and dry etching to open the seed windows through it. Before GeSn film deposition, this patterned substrate was dipped into diluted HF solution for several seconds to remove the natural oxide layer of the seed windows. A 160(200) nm amorphous GeSn film was deposited on Si_3_N_4_ layer using a MBE with a base pressure of 5 × 10^−8^ Pa at 80 °C. The Sn-content in amorphous GeSn film was about 15%. After deposition, the GeSn film was patterned into stripes with 2.2–2.7 μm widths and various lengths. 1 μm-thick SiO_2_ was deposited by plasma enhancement chemical vapor deposition (PECVD) at 300 °C to suppress the agglomeration and evaporation of the molten GeSn stripes. Rapid thermal annealing (RTA) was used to heat the samples to 930 °C for 1 s with a ramp up rate of 100 °C/s. Relative high cooling rate is about 60 °C/s which guarantees continuous growth of the GeSn stripe, especially at the end of stripe.

### Crystal orientation measurement

After the SiO_2_ was removed by HF, the crystal orientation of the GSOI stripe was characterized using EBSD (Nordlys max2, Oxford Instruments) with the scanning step of 100 nm.

### Sn-content measurement

The Sn-content and strain of the GeSn stripes were estimated using micro-Raman scattering spectra and TEM. The micro-Raman measurements were performed using Raman instrumentation (LabRAM HR800, HORIBA Jobin Yvon) at room temperature, with a 488 nm line Ar + laser and a Si photodetector. The spot size of the laser on the sample was approximately 1.5 μm. Cross-sectional TEM specimens were prepared using a dual-beam focused ion beam (FIB) apparatus (FEI Helios Nanolab 600). STEM and TEM images were recorded using a transmission electron microscope (FEI Tecnai G2 F20 S-Twin). EDX and SAED under the TEM model were employed to study the chemical composition and the structure of the GSOI stripe.

### Device fabrication

To study the optical and electrical characteristics of the GSOI, back-gate pseudo-MOSFETs and MSM photodetectors were fabricated. Firstly, the 1 μm SiO_2_ cover layer on the GeSn stripes was reduced to ~350 nm by dry etching. Next, electrode’s contact holes on the GeSn stripes were formed by HF wet etching the residual SiO_2_ capping layer. A 100/300 nm-thick Ni/Al electrode layer was formed on contact hole. Finally, Heads of the stripes were removed by dry etching to disconnect contact to Si substrate.

### Electrical measurement

The I-V and C-V characteristics of the devices were obtained using an Agilent B1500A semiconductor device analyzer.

### Optical responsivity measurement

The incident light was introduced by a tapered lensed fiber to the top surface of the device light-receiving area. The diameter of the incident light beam was about 4–7 μm, which was much larger than the light-receiving area of the GSOI.

## Additional Information

**How to cite this article**: Liu, Z. *et al*. Defect-free high Sn-content GeSn on insulator grown by rapid melting growth. *Sci. Rep.* **6**, 38386; doi: 10.1038/srep38386 (2016).

**Publisher's note:** Springer Nature remains neutral with regard to jurisdictional claims in published maps and institutional affiliations.

## Supplementary Material

Supplementary Dataset 1
