# Supplementary material for: Defect-free high Sn-content GeSn on insulator grown by rapid melting growth
Source: Sci Rep. 2016 Dec 12;6:38386. doi: 10.1038/srep38386 (PMC5150248; doi:10.1038/srep38386)
Supplement: Supplementary Dataset 1 [file srep38386-s1.doc]

Defect-free high Sn-content GeSn on insulator grown by rapid melting growth

Zhi Liu, Hui Cong, Fan Yang, Chuanbo Li, Jun Zheng, Chunlai Xue, Yuhua Zuo, Buwen Cheng*, Qiming Wang

State Key Laboratory on Integrated Optoelectronics, Institute of Semiconductors, Chinese Academy of Sciences, Beijing 100083, People’s Republic of China

*cbw@semi.ac.cn

This file contains:

1. Si-content distribution of the GSOI stripe with different lengths.

2. Sn-content distribution of the GSOI stripe with different lengths.

3. I-V characteristic of the devices with different average Sn-content.

4. Comparison of responsivity of GeSn top-illuminated photodetectors

5. Band structure calculations.

6. References

**1. Si-content distribution of the GSOI stripe with various lengths.**

The Si-content (*y*) of GSOI stripes can be calculated from the ratio between the integrated intensities of the Raman peaks corresponding to the Ge-Ge bonds (I_Ge-Ge_) and Ge-Si bonds (I_Si-Ge_) [^1^](#_ENREF_1) :

$y=\frac{\alpha}{\alpha+2I_{Ge-Ge}/I_{Ge-Si}}$ (1)

The parameter *α* is a constant which depends on the experimental conditions. We obtained *α* from the Raman spectra of many samples with various compositions of Ge-rich SiGe layers, in which the Ge-contents were calculated from *x*-ray diffraction measurements. In this way, we determined the coefficient *α*~1.4 for our experimental conditions. Fig. S1 shows the Si-content distribution of the GSOI stripe with different lengths. Si-Ge mixing and diffusion from Si seed into GSOI stripe are observed clearly. Lateral gradient in the Si-content is created along the GSOI stripes. This solidification temperature spatial gradient is the main driving force that caused the lateral growth of GSOI. Si-content is detected at front-part (~77%) of the GSOI near the Si seed. No Si-content is found at the rear-part (~23%) of the GSOI. This intense lateral Si diffusion is the typical feature of completely melting of the GeSn stripe. The diffusion of Si-atoms into melting Ge is very fast [^2^](#_ENREF_2).The diffusion length of Si-atoms even reaches 150 μm in the 175 μm-length GSOI. Moreover, in our previous Ge RMG work, such lateral Si diffusion only occurs when the Ge stripe is completely melting [^3^](#_ENREF_3).


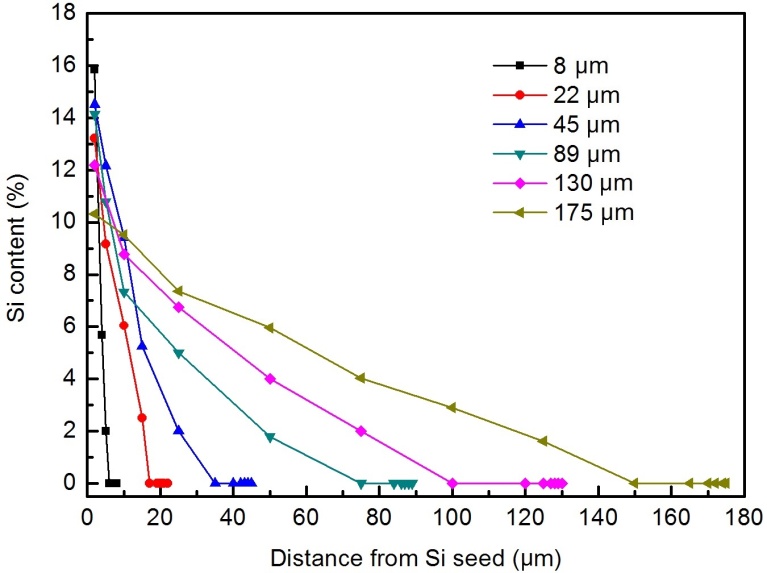


Fig. S1 Si-content distribution of the GSOI stripe with various lengths

**2. Sn-content distribution of the GSOI stripe with various lengths.**

Sn-content distribution of the GSOI stripe with various lengths are shown in Fig. S2. The highest Sn-content of the GSOI strong depends on the length of the GSOI stripe. The highest Sn-content raises as the length of GSOI increasing. When the length of the GSOI reaches 89 μm, the highest Sn-content is trend to saturation. The highest Sn-content at the end of the GSOI is about 14.4%, which is obtained from the GSOI with length larger than 89 μm. The Scheil equation is used to calculate the Sn-content distribution along the GSOI by the following expression [^4^](#_ENREF_4):

$x=x_{0}\left(d\right)k\left(1-d/L\right)^{k-1}$ (2)

where *k* (0.02) is the segregation coefficient of the Sn at the solid-Ge/liquid-Ge interface [^5^](#_ENREF_5), *x*_0_(*d*) is the average Sn-content of liquid-GeSn in different positions, *L* is the whole length of the stripe (included Sn metal), and *d* is the distance from the Si seed. Due to the most Ge atoms segregated in lateral growth process, the average Sn-content of liquid-GeSn increases at the position far from the Si seed. Thus, *x*_0_(*d*=0) is the initial Sn-content (0.15). At the end of GSOI, the x_0_(*d*=end) almost equal to 1. Fig. S2 also shows the Sn-content simulation curves of GSOIs with various length for comparison. The calculation results are in good agreement with the experimental results of GSOI stripes, especially at high Sn-content part. However, at the middle of the GSOI, a deviation between the experimental results and calculation results is observed. This deviation may be induced by the Si-Ge mixing, in which simple two-elements Scheil equation are not satisfied.


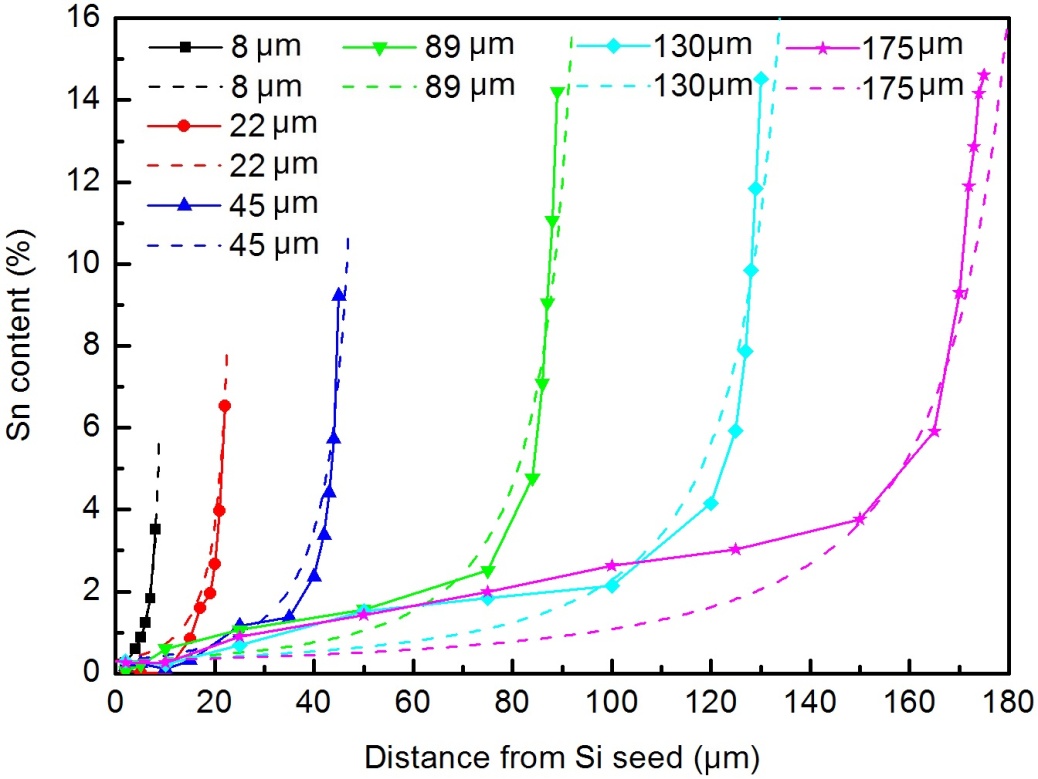


Fig. S2 Sn content distribution of the GSOI stripe with various lengths. Sn-content simulation curves of GSOIs with various length are shown in dash lines.

**3. I-V characteristic of the devices with different average Sn-content.**

Fig. S3(a) shows the I-V characteristic of the devices with different average Sn-content. The device exhibits an obvious rectifying behavior. The dark current increase as the average Sn-content increasing was observed clearly. A schematic of the device under bias voltage and a schematic of band structure of the device are shown in Fig. S3(b) and Fig. S3(c), respectively. Like the Fig. S3(b), the bias voltage was applied in the electrode which contact with the low Sn-content GSOI region. Thus, when the bias voltage is positive, the depletion region is formed on the high Sn-content GSOI region. when the bias voltage is negative, the depletion region is formed on the low Sn-content GSOI region. The current partly depends on the barrier height of the junction. The current at the positive bias voltage is larger than the that at negative bias voltage. Therefore, Schottky junction has different barrier height at GeSn/Ni contacts and the barrier is lower when GeSn layer has higher Sn concentration.

**
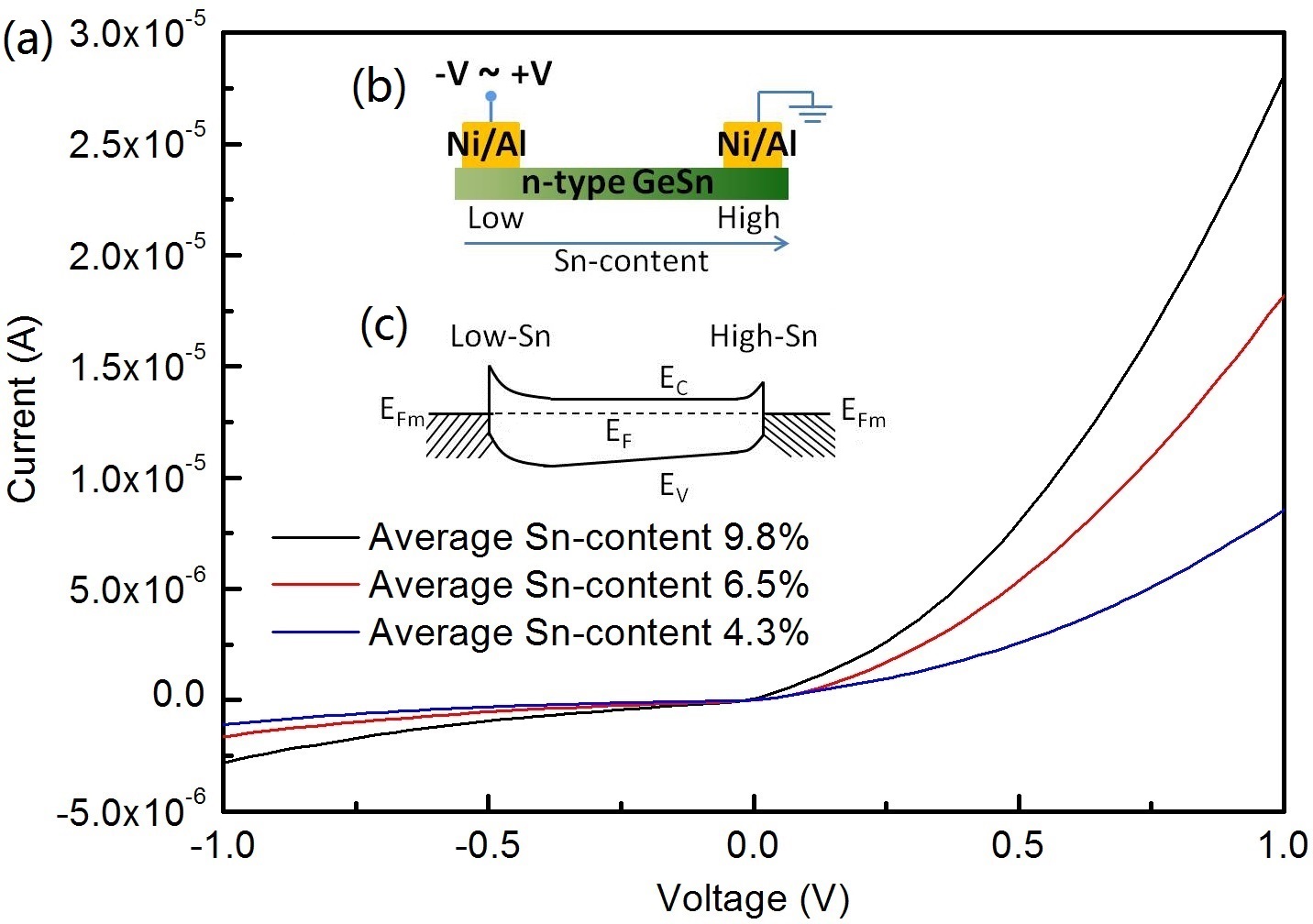
**

Fig. S3(a) I-V characteristic of the devices with different average Sn-content. (b) a schematic of the device under bias voltage. (c) a schematic of band structure of the device.

**4. Comparison of responsivity of GeSn top-illuminated photodetectors**

Table S1 shows comparison of responsivity of GeSn top-illuminated photodetectors at 300K. Pure Ge has no optical responsivity at 2000 nm. GeSn becomes to absorb the light at 2000 nm by direct band, When the Sn-content larger than 4%. As shown in Table S1, The responsivity of our GeSn device at 2000 nm is the highest for Si-based GeSn photodetector so far. The responsivity of top-illuminated photodetector depends on the thickness of the light absorption layer. Therefore, if compared with the GeSn photodetector with same thickness of absorption layer, the responsivity at 1550 nm also is the highest result for GeSn photodetector. The photodetectors with gain mechanism, such as avalanche photodetector and photoconductive photodetector, are not suitable for comparison. Thus, they are not listed in the Table S1.

**Table S1 Comparison of responsivity of GeSn top-illuminated photodetectors at 300K**

| **Materials and thickness** | **Responsivity at 1.55μm** | | **Responsivity at ~2μm** |
| --- | --- | --- | --- |
| 820nm Ge_0.97_Sn_0.03_+240nm i-Ge[^6^](#_ENREF_6)  750nm Ge_0.964_Sn_0.036_ on Ge Sub.[^7^](#_ENREF_7)  310nm Ge_0.96_Sn_0.04_[^8^](#_ENREF_8)  360nm Ge_0.95_Sn_0.05_+350nm i-Ge[^9^](#_ENREF_9)  200nm Ge_0.93_Sn_0.07_+700nm p-Ge[^10^](#_ENREF_10)  200nm Ge_0.9_Sn_0.1_+700nm p-Ge[^10^](#_ENREF_10)  This work: 200nm Ge_0.91_Sn_0.09_ | 0.23 A/W  0.6 A/W  0.181 A/W  0.178 A/W  0.3 A/W  0.19 A/W  0.236 A/W | Cutoff at 1.8μm  0.05 A/W at 2μm  0.01 A/W at 2μm  0.017 A/W at 1.88μm  Unknown  Unknown  0.154 A/W at 2μm | |

**5. Band structure calculations**

The bandgap of GeSn alloy without strain is calculated from the $E_{g}^{Ge}$, $E_{g}^{\alpha-Sn}$, and bowing parameter $b^{GeSn}$ by following equation:

$E_{g}^{GeSn}\left(x\right)=E_{g}^{Ge}(1-x)+E_{g}^{\alpha-Sn}x-b^{GeSn}x\left(1-x\right)$ (3)

Due to the tensile strain, the light hole (LH) valence band is shifted towards higher energies as compared to the heavy hole (HH) valence band. The band structure of the GeSn alloy under strain was calculated by the deformation potentials theory [^11^](#_ENREF_11). The parameters of GeSn alloy are obtained by linear interpolation, and Table S2 shows these parameters of bulk Ge and α-Sn, respectively. Fig. S3 shows the calculational bandgap for the 0.15% tensile strained GeSn with Sn-contents of 1.9%, 4.3 %, and 9.8 %, respectively. The bandgap of the GeSn layers with Sn-contents of 1.9%, 4.3 % is corresponding to the indirect bandgap edge absorption and direct bandgap edge absorption at 0.62 eV (2000 nm) by transition between the LH valence band and conduction band.

**Table S2 Constants used for calculation of GeSn energy bandgap.(eV)**[**^12-14^**](#_ENREF_12)

| **Material** | **E_v,av_** | **a_v_** | **a_c_ ^Γ^** | **a_c_^L^** | **Δ_0_** | **b** | **E_g_ ^Γ^** | **E_g_^L^** | **b^GeSn^ _Γ_** | **b^GeSn^ _L_** |
| --- | --- | --- | --- | --- | --- | --- | --- | --- | --- | --- |
| **Ge** | -6.35 | 1.24 | -8.24 | -1.54 | 0.3 | -2.86 | 0.804 | 0.660 | 2.55 | 0.89 |
| **α-Sn** | -5.66 | 1.55 | -5.33 | -0.34 | 0.8 | -2.7 | -0.413 | 0.092 |


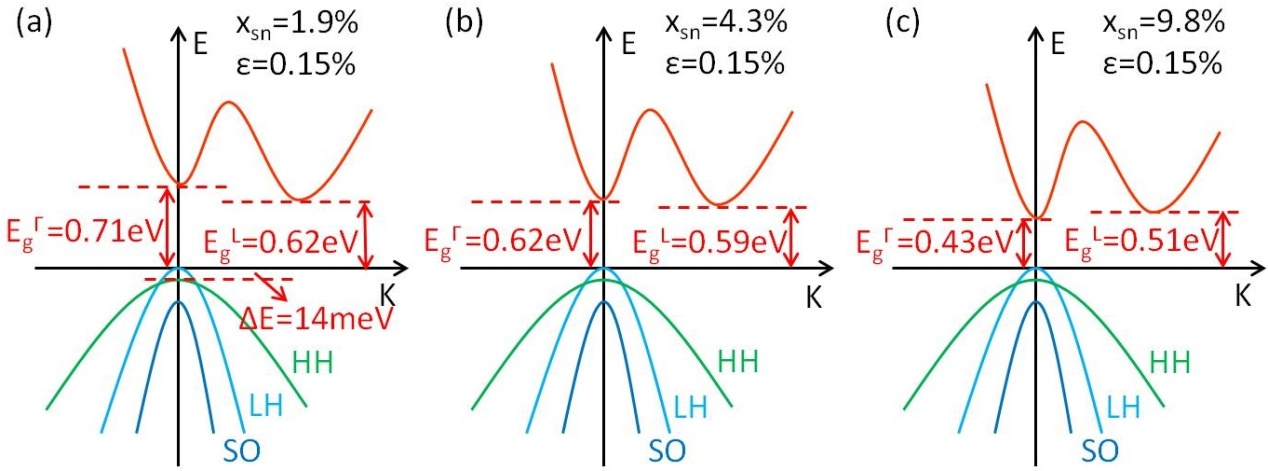


Fig. S3 Bandgaps at 300K calculated for the 0.15% tensile strained GeSn with Sn contents of 1.9%, 4.3 %, and 9.8 %, respectively.

**6. References**

1. Mooney P. M., Dacol F. H., Tsang J. C. & Chu J. O. Raman scattering analysis of relaxed Ge_x_Si_1−x_ alloy layers. *Appl. Phys. Lett.* **62**, 2069-2071 (1993).

2. Bai X., Chen C.-Y., Griffin P. B. & Plummer J. D. Si incorporation from the seed into Ge stripes crystallized using rapid melt growth. *Appl. Phys. Lett.* **104**, 052104 (2014).

3. Liu Z.*, et al.* Lateral growth of single-crystal Ge on insulating substrate using amorphous Si seed by rapid melting growth. *Thin Solid Films* **597**, 39-43 (2015).

4. Scheil E. Bemerkungen zur schichtkristall bildung. *Z. Metallkd.* **34**, 70-72 (1942).

5. Kurosawa M., Tojo Y., Matsumura R., Sadoh T. & Miyao M. Single-crystalline laterally graded GeSn on insulator structures by segregation controlled rapid-melting growth. *Appl. Phys. Lett.* **101**, 091905 (2012).

6. Su S.*, et al.* GeSn p-i-n photodetector for all telecommunication bands detection. *Opt. Express* **19**, 6408-6413 (2011).

7. Zhang D.*, et al.* High-responsivity GeSn short-wave infrared p-i-n photodetectors. *Appl. Phys. Lett.* **102**, 141111 (2013).

8. Oehme M.*, et al.* GeSn p-i-n detectors integrated on Si with up to 4% Sn. *Appl. Phys. Lett.* **101**, 141110 (2012).

9. Dong Y.*, et al.* Suppression of dark current in germanium-tin on silicon p-i-n photodiode by a silicon surface passivation technique. *Opt. Express* **23**, 18611-18619 (2015).

10. Pham T.*, et al.* Systematic study of Si-based GeSn photodiodes with 2.6μm detector cutoff for short-wave infrared detection. *Opt. Express* **24**, 4519-4531 (2016).

11. Van de Walle C. G. Band lineups and deformation potentials in the model-solid theory. *Phys. Rev. B* **39**, 1871-1883 (1989).

12. Yin W.-J., Gong X.-G. & Wei S.-H. Origin of the unusually large band-gap bowing and the breakdown of the band-edge distribution rule in the Sn_x_Ge_1−x_ alloys. *Phys. Rev. B* **78**, 161203 (2008).

13. Chang S. W. & Chuang S. L. Theory of optical gain of Ge-Si_x_Ge_y_Sn_1-x-y_ quantum-well lasers. *Ieee J. Quantum Elect.* **43**, 249-256 (2007).

14. Yuan-Hui Z., Qiang X., Wei-Jun F. & Jian-Wei W. Theoretical gain of strained GeSn_0.02_Ge_1-x-y_,Si_x_Sn_y_, quantum well laser. *J. Appl. Phys.* **107**, 073108 (2010).
